# Changes in breathing pattern during severe hypothermia and autoresuscitation from hypothermic respiratory arrest in anesthetized mice

**DOI:** 10.14814/phy2.15139

**Published:** 2021-12-12

**Authors:** Saki Taiji, Takashi Nishino, Hisayo Jin, Norihiro Shinozuka, Natsuko Nozaki‐Taguchi, Shiroh Isono

**Affiliations:** ^1^ Department of Anesthesiology Graduate School of Medicine Chiba University Chiba Japan; ^2^ Department of Anesthesiology Chibaken Saiseikai Hospital Chiba Japan

**Keywords:** adult mice, anesthetics, breathing pattern, hypothermia, respiratory arrest

## Abstract

Some evidence suggests that both hypothermia and anesthesia can exert similar effects on metabolism and ventilation. This study examined the synergistic effects of anesthesia and hypothermia on ventilation in spontaneously breathing adult mice under three different conditions, that is, (1) pentobarbital group (*n* = 7) in which mice were anesthetized with intraperitoneal pentobarbital of 80 mg/kg, (2) sevoflurane‐continued group (*n* = 7) in which mice were anesthetized with 1 MAC sevoflurane, and (3) sevoflurane‐discontinued group (*n* = 7) in which sevoflurane was discontinued at a body temperature below 22˚C. We cooled mice in each group until breathing ceased and followed this with artificial rewarming while measuring changes in respiratory variables and heart rate. We found that the body temperature at which respiration arrested is much lower in the sevoflurane‐discontinued group (13.8 ± 2.0˚C) than that in the sevoflurane‐continued group (16.7 ± 1.2˚C) and the pentobarbital group (17.0 ± 1.4˚C). Upon rewarming, all animals in all three groups spontaneously recovered from respiratory arrest. There was a considerable difference in breathing patterns between sevoflurane‐anesthetized mice and pentobarbital‐anesthetized mice during progressive hypothermia in terms of changes in tidal volume and respiratory frequency. The changes in the respiratory pattern during rewarming are nearly mirrored images of the changes observed during cooling in all three groups. These observations indicate that adult mice are capable of autoresuscitation from hypothermic respiratory arrest and that anesthesia and hypothermia exert synergistic effects on the occurrence of respiratory arrest while the type of anesthetic affects the breathing pattern that occurs during progressive hypothermia leading to respiratory arrest.

## Graphical abstract

Adult mice are capable of autoresuscitation from respiratory arrest, regardless of the presence or type of anesthetics. Anesthesia increases the body temperature at which respiratory arrest occurs. The type of anesthetic affects the breathing pattern that occurs during progressive hypothermia leading to respiratory arrest.

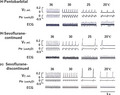

## 1 INTRODUCTION

Most species of adult mammals cannot tolerate body temperatures below 15–20˚C; the temperatures at which cessation of respiratory activity takes place. And unless there is ventilatory assistance the animals eventually die during artificial rewarming (Adolph, 1951; Adolph & Goldstein, 1959; Hamilton, 1937; Hill & Eshuis, 1988). In contrast, most species of neonatal mammals and some adults of hibernating species of mammals can tolerate much lower body temperatures. And artificial rewarming after respiratory arrest leads to spontaneous recovery from this respiratory arrest without any post‐hypothermic sequelae. This is termed “autoresuscitation from hypothermic respiratory arrest” (Adolph, 1948a, 1948b, 1951; Corcoran et al., 2012; Tattersal & Milsom, 2003). Although the mechanisms of hypothermic respiratory arrest and subsequent autoresuscitation in mammals remain to be clarified, a similar effect of hypothermia on respiration has been reported in studies using anesthetized animals in which focal cooling of the brain stem was performed (Cherniack et al., 1979; Kiley et al., 1984, 1985; Millhorn et al., 1982) and in studies using an isolated in vitro brainstem preparation (Mellen et al., 2002; Zimmer & Milsom, 2004). The results of these studies suggest that hypothermic respiratory arrest is probably due to the reversible failure of the rhythm‐generating network in the brainstem and that as long as sufficient energy and oxygen are supplied to the brainstem, the ability of the brainstem to generate spontaneous respiratory activity remains intact despite severe hypothermic stress and biological insult.

Most of the in vivo hypothermic studies on respiration were performed in conscious animals and only a few studies dealt with the effects of hypothermia on the breathing pattern in anesthetized animals (Gautier & Gaudy, 1986; Kiley et al., 1984, 1985; Osborne & Milsom, 1993). Although there is much evidence to show that breathing patterns are influenced by many factors such as age, size, species, and the choice of anesthetics (Milic‐Emili, 1977), to our knowledge, no study has dealt with the influence of anesthesia on the ability to autoresuscitate from hypothermic respiratory arrest in adult mammals that do not hibernate. There is some evidence to show that light anesthesia with halothane improves the ability to survive hypothermia in hibernating mammals (Volkert & Musacchia, 1976). In anesthetized animals, the inhibitory effects of hypothermia on respiratory activity might be magnified by the synergistic effects of anesthesia since the effects of anesthesia on metabolism and ventilation are to a certain extent analogous to the effects of hypothermia (Hagerdal et al., 1978; Lin et al., 2014; Mielck et al., 1999; Osborne & Milsom, 1993; Steen et al., 1983).

We hypothesized that (1) anesthesia and hypothermia could synergistically suppress brainstem mechanisms and thereby could enhance the development of respiratory arrest and (2) changes in respiratory patterns during progressive hypothermia may vary depending on the choice of anesthetic. The mouse is the most predominant test species in recent biomedical research. Since it has been shown that the adult laboratory mice do not hibernate, but they exhibit a short‐term hypometabolic state known as daily torpor (Sunagawa & Takahashi, 2016; Swoap & Gutilla, 2009), we framed an additional hypothesis that the anesthetized adult mice could show the ability to autoresuscitate from hypothermic respiratory arrest, as long as the most suitable condition for resuscitation is provided during artificial rewarming. In this study, we investigated changes in breathing patterns during progressive hypothermia leading to respiratory arrest in spontaneously breathing adult mice anesthetized with two different types of anesthetics, that is, sevoflurane and pentobarbital. We also investigated the process of autoresuscitation from hypothermic respiratory arrest during rewarming in these mice.

## 2 METHODS

### 2.1 Ethical approval

The present experiments were performed under the “Guiding Principles for the Care and Use of Animals in the field of Physiological Sciences” recommended by the Physiological Society of Japan. All of the experimental protocols were approved by the Committee on Animal Research at the University of Chiba (animal protocol number 2‐206).

### 2.2 Animals

Twenty‐one adult male inbred mice (C57BL/6, Harlan, the Netherlands), housed in animal facilities at Chiba University, aged 28–36 weeks and weighing between 31 and 39 g were used. They were maintained in a conventional housing system on a 12:12 h light–dark cycle and a room temperature of 21–23˚C with unlimited access to food and water until the time of the experiment.

### 2.3 Experimental protocol

At the beginning of the experiments, the animals were initially anesthetized either with intraperitoneal injection of pentobarbital sodium (80 mg/kg) (pentobarbital group; *n *= 7) or with 5%–7% of sevoflurane (*n* = 14). In all anesthetized mice, tracheal intubation was performed with a 22G intravenous catheter using SIAC (supraglottic intubation‐aid conduit) in the supine position (Jin et al., 2019). The details of anesthetic induction with sevoflurane and tracheal intubation were described elsewhere (Nishino et al., 2020). After tracheal intubation, all of the animals breathed spontaneously through a T‐piece circuit into which oxygen was delivered at a flow rate of 0.5 L/min. The sevoflurane‐anesthetized mice were divided into two subgroups: (1) sevoflurane‐continued group (*n *= 7) and (2) sevoflurane‐discontinued group (*n* = 7). All of the sevoflurane‐anesthetized mice received 3.2%–3.3% sevoflurane (1 MAC: minimum alveolar concentration) for 10–15 min before the start of progressive hypothermia (baseline period 1) while the inspired concentration of sevoflurane was continuously monitored with an anesthetic gas monitor (Multigas Unit AG‐920R, Nihon Kohden). In both the sevoflurane‐continued group and the sevoflurane‐discontinued group, sevoflurane anesthesia was maintained at 1 MAC during progressive hypothermia starting at normothermia, that is, a body temperature of 36.0–36.5˚C and reducing the body temperature to 22˚ C. Then, in the sevoflurane‐continued group inhalation of 1 MAC sevoflurane was continued whereas in the sevoflurane‐discontinued group, administration of sevoflurane was discontinued at and below the body temperature of 22˚C so that the condition of anesthesia was maintained by hypothermia alone (cold narcosis) during progressive hypothermia below 20˚C. In these animals, the adequate level of “cold narcosis” was determined by pinching the tail and observing no flinching or withdrawal.

The body temperature of the animals was measured continuously with a rectal temperature monitor (AD‐1687, A&D Co LTD). Animals were cooled and maintained cool with an electric air cooling fan (Machito‐ZXHT LSF‐088, Quandong) and were rewarmed with a hairdryer (Ione‐Tescom TID 724) and/or a heating lamp.

Surface cooling of the animal to progressively lower body temperature was initialized by directing a stream of cooled air (6–10˚C) over the animal while the ambient temperature around the animal was continuously monitored with a digital thermometer (DT‐300, Inter Medical Co LTD). Cooling of body temperature was continued (at the rate of approximately 0.5˚C/min) until respiratory activity ceased completely. After the onset of respiratory arrest, at least 1 min was allowed to elapse after which rewarming was started. The heating lamp and the hairdryer were fixed at such a distance from the animal so that the ambient temperature around the animal did not exceed 43˚C. The administration of sevoflurane (1 MAC) was resumed at a body temperature of 22˚C during the rewarming process in mice in the sevoflurane‐discontinued group. The rewarming procedure continued until the body temperature reached the normothermic level which was then maintained for 10–15 min (baseline period 2).

The precise measurement of ventilatory airflow in spontaneously breathing mice was made by a hand‐made pneumotachograph attached to the tracheal cannula. Tidal volume (*V*_T_) was obtained by electrical integration of the inspired airflow signal. Immediately before and after each experiment, the pneumotachograph was calibrated with fixed volumes (0.1, 0.2, and 0.4 ml) of carrier gas (100% O_2_) at a rate of 60 strokes/min using a 1 ml syringe (SS‐01T, Terumo). Tracheal pressure (Ptr) was measured with a pressure transducer (TruWave pressure transducer, Edwards Lifesciences, CA, USA) with an amplifier (AP‐601G, Nihon Kohden). ECG was monitored through soft surface electrodes attached at the right front paw, left front paw, and left hind limb. Heart rate (HR) was determined from the ECG recording.

During the experiments, airflow, tidal volume, tracheal pressure, and ECG all were recorded on a thermal array recorder (Omniace RT 3424; NEC) and stored on a Magneto Optical disk for later analysis of the data using a computer program (Omniwin RT34704; NEC). After the experiments, the animals were extubated and returned to their cages in the animal facility.

All of the animals were observed for the following 3 days for signs of post‐experimental complications including neurological damages (A physical examination and behavioral assessment of the animals were performed by one of the authors (H.J.) at 10 am each morning for 3 days after the experiment).

### 2.4 Data analysis

Commercial software was used for statistical analyses (SigmaPlot 11.2, Systat Software, Inc.). The breathing pattern was analyzed in terms of tidal volume (*V*_T_), respiratory frequency (*f*_R_), inspiratory time (*T*_I_), expiratory time (*T*_E_), mean inspiratory flow (*V*_T_/*T*_I_), total breath duration (*T*_tot_), inspiratory duty cycle (*T*_I_/*T*_tot_), and minute ventilation (*V*_I_) which is defined as the product of *V*_T_ and *f*_R_.

To clarify the effects of progressive cooling and rewarming, five target body temperatures at approximately 5˚C intervals were tentatively set. These were 36.0–36.5˚C (normothermia), 30˚C (mild hypothermia), 25˚C (moderate hypothermia), 20˚C (severe hypothermia), and below 20˚C at respiratory arrest (profound hypothermia). The period of profound hypothermia includes the period of respiratory arrest (RA), the pre‐RA period, the last breath, the first breath, and the post‐RA period. The pre‐RA period and the post‐RA period correspond to the periods of approximately 2 min before and 2 min after the hypothermic respiratory arrest, respectively. All respiratory variables and HR were measured over a 1‐min period immediately after a targeted body temperature was reached, and each average value for each temperature was treated as a single measurement for purposes of analysis. The whole experimental period was divided into three different phases, that is, (1) the cooling phase from normothermia down to 20˚C, (2) the profound hypothermic phase at the body temperature of below 20˚C immediately before and after respiratory arrest, which include the last and first breaths, and (3) the rewarming phase from 20˚C back to normothermia.

Since breathing was considered to have arrested when no breaths were taken for over 60 s, when analyzing the data of the last breath during the profound hypothermic phase, the duration of *T*_E_ and the respiratory frequency of the last breath were recorded as 60 s and 1/min, respectively.

In order to see the approximate effects of cooling and rewarming on the circulatory and respiratory control systems, a linear regression analysis was performed to assess the relationship between HR and *V*_I_ during cooling and rewarming in all three experimental groups. Since some respiratory variables failed normality and exhibited unequal variances across the wide range of body temperatures, a repeated‐measures ANOVA on ranks (Friedman test) followed by the Dunnett's test (post hoc analysis) was performed in this study to distinguish within‐group differences in respiratory variables during the continuous cooling phase, the profound hypothermic phase, and the rewarming phase in each treatment group.

In these analyses, the values of respiratory variables obtained from the baseline 1 period served as the control values during cooling and rewarming, whereas during profound hypothermia the values of respiratory variables obtained from the last breath served as the control values. For comparisons of respiratory variables at each targeted temperature during cooling and rewarming and at each classified period during profound hypothermia among the three experimental groups, one‐way ANOVA on ranks and post hoc analysis with the Tukey's test were used to test for significant differences in each respiratory variable. One‐way ANOVA (parametric method) was also used to test significant differences in the rates of cooling and rewarming and the temperatures at which respiratory arrest occurred among the three different groups. When the data were analyzed with a nonparametric method, the data were expressed as median (interquartile range) whereas when data were analyzed with a parametric method, the data were presented as mean ± SD. *p *< 0.05 was considered as significant.

## 3 RESULTS

### 3.1 Changes in body temperature during cooling and rewarming

The animals in all three groups were cooled at the same rate to a body temperature at which respiration arrested. Respiratory arrest took longer to occur in some animals because they had to be cooled to lower temperatures in order for respiration to cease. Total time to respiratory arrest took between 32 and 71 min. One minute after respiratory arrest, rewarming was started. Table 1 shows data on select variables during cooling and rewarming. In all three groups, the rate of cooling was similar when the body temperature decreased from normothermia until respiratory arrest. A longer time was required to reach the body temperature at which respiration arrested in the sevoflurane‐discontinued group, compared with other two groups that had continuous anesthesia. This is because this temperature was lower than in both of the anesthetized groups. With the start of rewarming, the body temperature gradually returned to normothermic levels with a similar rate of rewarming in all three groups.

**TABLE 1:** Data on select variables during cooling and rewarming

Anesthetic condition	Pentobarbital	Sevoflurane‐continued	Sevoflurane‐discontinued
Rate of cooling (˚C/min)	0.51 ± 0.10	0.49 ± 0.06	0.42 ± 0.09
Cooling duration (min)	38.7 ± 8.1	40.1 ± 6.7	56.1 ± 15.3* ^,^ ^#^
Rate of rewarming (˚C/min)	0.70 ± 0.08	0.63 ± 0.17	0.65 ± 0.15
Rewarming duration (min)	27.0 ± 1.8	30.1 ± 4.5	35.6 ± 11.4
BT at respiratory arrest (˚C)	17.0 ± 1.4	16.7 ± 1.2	13.8 ± 2.0* ^,^ ^#^
Duration of respiratory arrest (min)	3.7 ± 0.8	2.8 ± 0.9	4.9 ± 2.8
BT at which breathing restarted (˚C)	17.9 ± 1.2	17.3 ± 1.7	15.4 ± 1.9*
Survival rate (%)	100	100	100

Values are mean ± SD.

BT, body temperature.

* *p *< 0.05, compared with the values of pentobarbital group.

# *p *< 0.05, compared with the values of sevoflurane‐continued group.

### 3.2 Changes in HR and *V*_I_ during the cooling and rewarming

**FIGURE 1:**
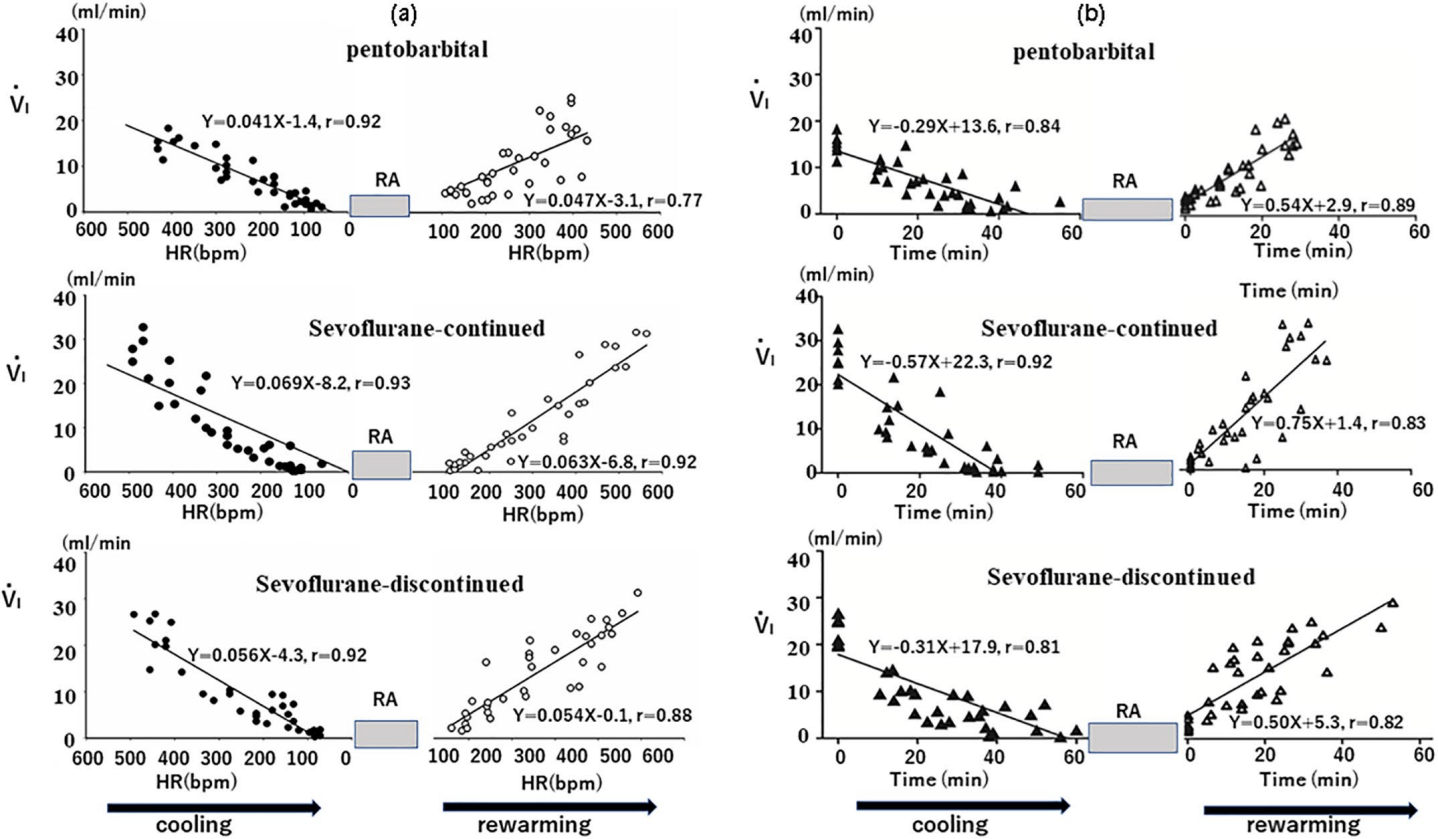
Relationships for HR (heart rate) versus minute ventilation (*V*_I_) (a) and for time versus *V*_I_ (b) during cooling and rewarming in three experimental groups. RA, respiratory arrest

In all animals, regardless of the type of anesthetic, the values of heart rate (HR: in bpm, beats per min) and minute ventilation (*V*_I_) progressively decreased roughly in a linear fashion during cooling with time until the time of respiratory arrest and increased progressively when body temperature elevated with time during rewarming from the temperature at respiratory arrest to normothermia. Figure 1 shows the relationships for HR (beats/min) versus *V*_I_ (ml/min) and for time (s) versus *V*_I_ in mice of the three different groups. There was a significant correlation between HR and *V*_I_ and between time and *V*_I_ during cooling and rewarming in all three groups. In most mice in all three groups, the heart continued to beat during the period of respiratory arrest, however, in two mice sudden irregularities in ECG tracings resembling ventricular fibrillation appeared. The start of rewarming always brought these irregular ECG tracings back to regular ECG tracings. Although it was possible that hypothermia caused not only respiratory arrest but also cardiac arrest in these few mice, there is distinct possibility that the abnormal ECG tracings were brought about by ECG artifacts. All mice in all of the experiments survived the cooling and rewarming phases of the experiment.

### 3.3 Detailed analysis of breathing pattern

#### 3.3.1 Effect of cooling

**FIGURE 2:**
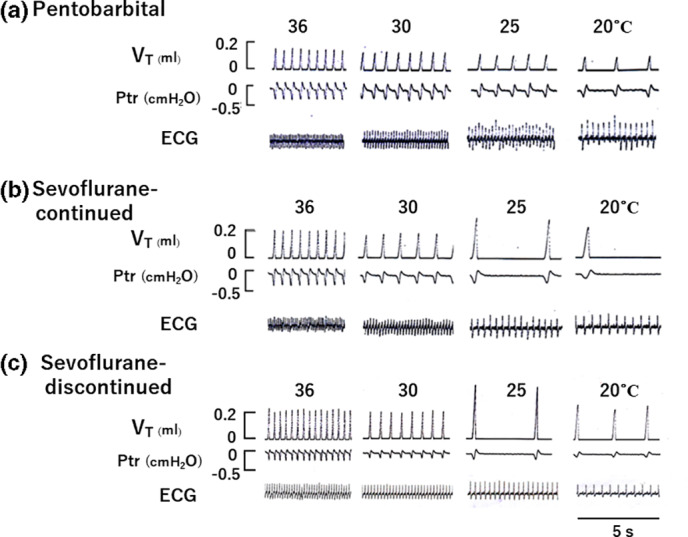
Experimental recordings illustrating changes in breathing patterns and ECG in response to decreases in body temperature in three different groups. (a) pentobarbital group; (b) sevoflurane‐continued; (c) sevoflurane‐discontinued group. *V*_T_, tidal volume; Ptr, tracheal pressure

Experimental records illustrating changes in breathing pattern during progressive cooling in the three different groups are shown in Figure 2. These recordings clearly show that changes in breathing pattern during cooling are quite different among three different groups, as described in more detail later.

**FIGURE 3:**
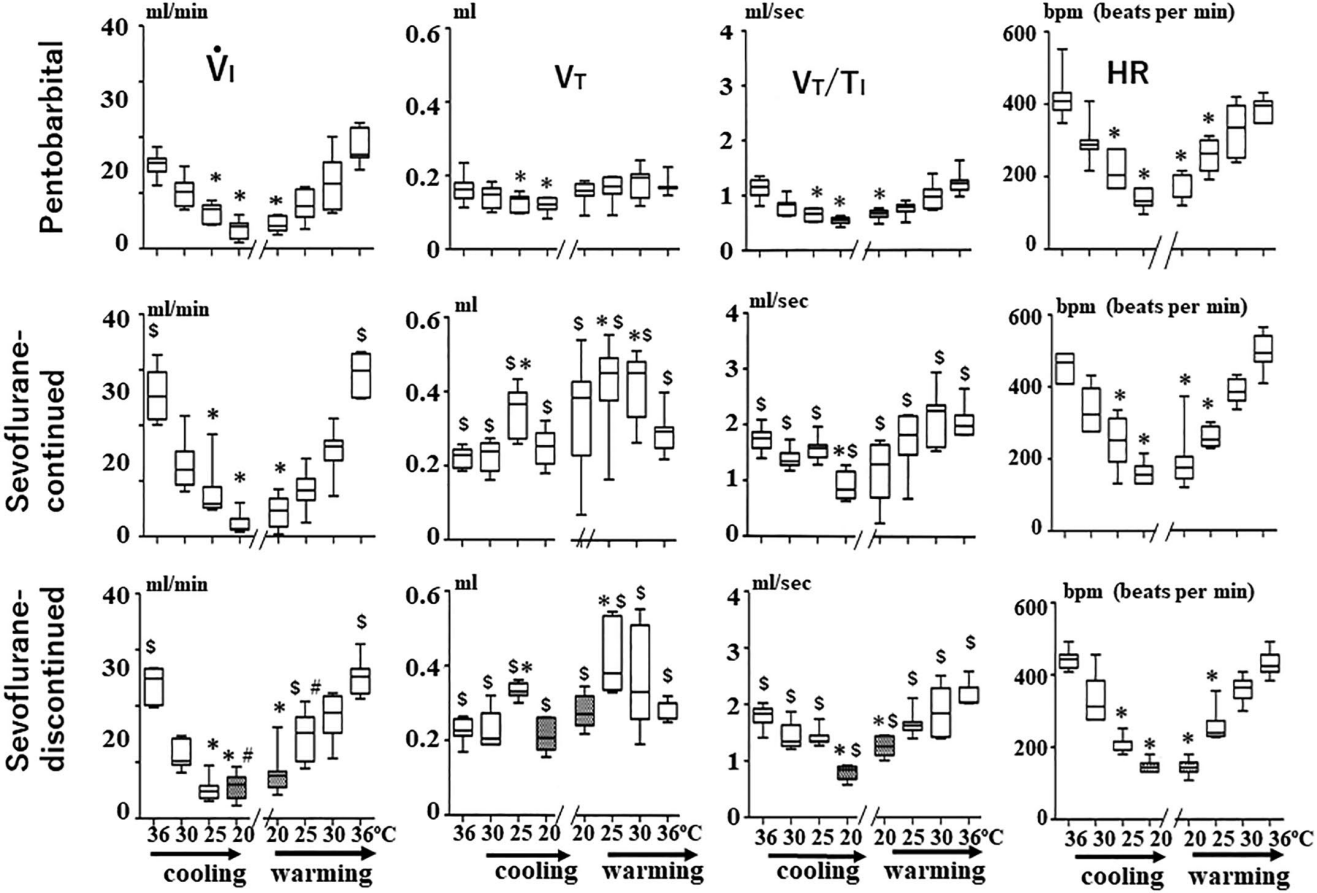
Box plots of changes in *V*_I_, *V*_T_, *V*_T_/*T*_I_, and HR during cooling and rewarming. The line in the middle of the box indicates the median, the upper and lower ends of the box indicate the upper and lower quartiles, and the upper and lower ends of the error bar indicate the maximum and minimum. The shaded box plot shows the values obtained during the non‐sevoflurane inhalation period. *p < 0.05, compared with the control values at normothermia; ^$^ *p *< 0.05, compared with the corresponding values in the pentobarbital groups; and ^#^ *p* < 0.05, compared with the corresponding values in the sevoflurane‐continued group

As shown in Figure 3, the control values of *V*_I_, *V*_T_, and *V*_T_/*T*_I_, at 36ºC in the pentobarbital group were significantly smaller than those in the sevoflurane‐continued group and in the sevoflurane‐discontinued group. These results suggest that ventilatory activity in the pentobarbital group was more depressed than that in the sevoflurane‐continued group and the sevoflurane‐discontinued group during the baseline period before the start of cooling.

**FIGURE 4:**
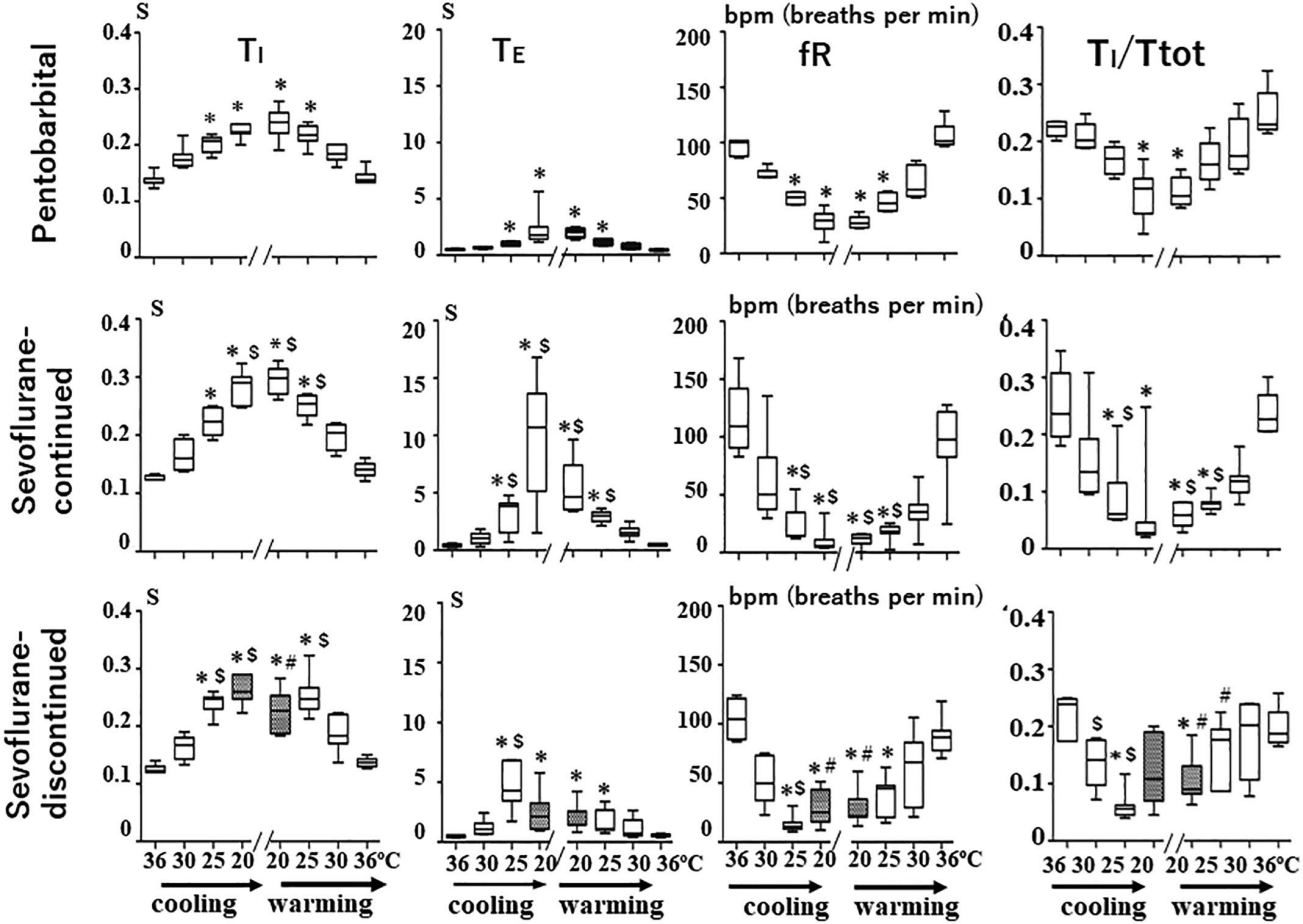
Box plots of changes in *T*_I_, *T*_E,_ *f*_R_, and *T*_I_/*T*_tot_ during cooling and rewarming. The box plot data show the median, interquartile range, minimum, and maximum values. *p < 0.05, compared with the control values at normothermia; ^$^ *p *< 0.05, compared with the corresponding values in the pentobarbital groups; ^#^ *p *< 0.05, compared with the corresponding values in the sevoflurane‐continued group

Figure 4 shows that in all three experimental groups, the values of breathing frequency (*f*_R_: in bpm, breaths per min) decreased in a roughly linear fashion between a body temperature of 36–25˚C.

In all three groups, the decrease in *f*_R_ during cooling depends on the prolongation of both *T*_I_ and *T*_E_. However, the changes in *T*_E_ were much more prominent than those of *T*_I_ with the progressive cooling of body temperature, particularly in mice anesthetized with sevoflurane (Figure 4).

Comparisons of the *f*_R_ values among the three different groups show that there was no significant difference between the values of *f*_R_ at 36 and 30˚C (Figure 4). However, the values of *f*_R_ at 25˚C in the sevoflurane‐continued group and the sevoflurane‐discontinued group were significantly lower than those in the pentobarbital group, whereas the values of *f*_R_ at 20˚C in the sevoflurane‐continued group were significantly lower than those in the pentobarbital group and the sevoflurane‐discontinued group. Concomitantly, the values of *T*_I_/*T*_tot_ in the sevoflurane‐continued group and the sevoflurane‐discontinued group at 25˚C were significantly smaller than those in the pentobarbital group, which can be attributed to the prominent prolongation of *T*_E_ (Figure 4).

Regarding changes in *V*_T_ during cooling, detailed analyses revealed that there were considerable differences in the values of *V*_T_ during cooling between mice anesthetized with pentobarbital and those anesthetized with sevoflurane including both the sevoflurane‐continued group and the sevoflurane‐discontinued group. Unlike in sevoflurane‐anesthetized mice, in pentobarbital‐anesthetized mice, there was a gradual, very small, and progressive decrease in *V*_T_ during continuous cooling from 36 to 20˚C (Figures 2 and 3). The values of *V*_T_/*T*_I_ also decreased progressively with the concomitant decrease in *V*_T_ during cooling in the pentobarbital‐anesthetized mice (Figure 3).

On the other hand, in sevoflurane‐anesthetized mice *V*_T_ did not change much during the early stages of cooling but when the body temperature reached below 28˚C, *V*_T_ gradually increased with a concomitant decrease in *f*_R_ and when the body temperature reached to near 25˚C, breathing patterns were characterized by augmented breaths consisting of large *V*_T_ and slow frequency breaths with respiratory mandibular movements (Figures 2, 3, 4). Despite this considerable increase in *V*_T_, however, there was no significant increase in *V*_T_/*T*_I_ at the body temperature of 25˚C. With further decreases in the body temperature in mice of both the sevoflurane‐continued group and the sevoflurane‐discontinued group, the elevated *V*_T_ decreased gradually, returning to normothermic values (Figure 3). In these mice, the values of *V*_T_/*T*_I_ at 20˚C were significantly lower than the values of *V*_T_/*T*_I_ at 36˚C (normothermia). The *V*_T_ and *V*_T_/*T*_I_ values of sevoflurane‐anesthetized mice during the cooling from 36°C to 20°C were always larger than those of pentobarbital‐anesthetized mice at the corresponding temperatures (Figure 3).

#### 3.3.2 Effect of rewarming

In the pentobarbital‐anesthetized group, changes in breathing pattern during rewarming roughly mirrored the changes observed during cooling. Thus, in this group during rewarming increases in *f*_R_, *V*_T_/*T*_I_, and *T*_I_/*T*_tot_ without a remarkable change in *V*_T_ were clearly observed (Figures 3 and 4). In this context, the increase in *V*_I_ during rewarming was mainly due to the increase in *f*_R_ (Figure 4).

In both the sevoflurane‐continued and sevoflurane‐discontinued groups, *V*_T_ increased and peaked with increasing body temperatures and was maximal during rewarming while causing augmented between 25 and 30˚C (Figure 3). These findings are in contrast to the observation made in the pentobarbital group but mirrored the findings seen during cooling, which showed remarkable increases in *V*_T_ at 25˚C. When the body temperature returned to normothermic levels (baseline period 2), the values of *V*_I,_ *V*_T_, and *V*_T_/*T*_I_ in the sevoflurane‐anesthetized mice were significantly higher than those in the pentobarbital‐anesthetized mice (Figure 3).

### 3.4 Changes in respiratory variables immediately before and after hypothermic respiratory arrest

The body temperatures at which breathing arrested in all three different groups are shown in Table 1. The values in pentobarbital group (17.0 ± 1.4˚C) and in sevoflurane‐continued group (16.7 ± 1.2˚C) were significantly higher than those in sevoflurane‐discontinued group (13.8 ± 2.0˚C) (*p *< 0.05), whereas there is no significant difference in temperatures at respiratory arrest between the pentobarbital group and the sevoflurane‐continued group. There were no significant differences in the values of duration of respiratory arrest among the three different groups (Table 1). The values of body temperature at which breathing re‐started during rewarming are also shown in Table 1. The values of body temperature at which breathing re‐started in the sevoflurane‐discontinued group were significantly lower than those in the pentobarbital group and the sevoflurane‐continued group (*p *< 0.05), whereas there is no significant difference in the values of body temperature at which breathing re‐started between the pentobarbital group and the sevoflurane‐continued group.

**FIGURE 5:**
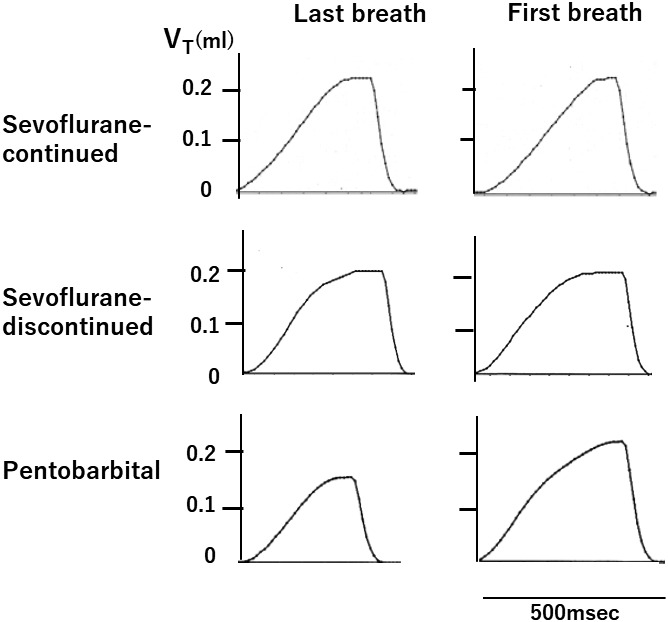
Representative traces showing a difference between the first breath and the last breath in three different groups

**FIGURE 6:**
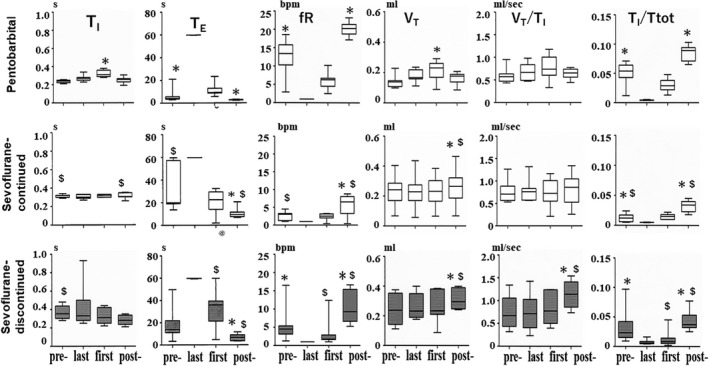
Changes in respiratory variables during profound hypothermia. The box plot data show the median, interquartile range, minimum, and maximum values. The shaded box plot shows the values obtained during the non‐sevoflurane inhalation period. pre‐, pre‐RA period; last, last breath; first, first breath; post‐, post‐RA period. *p < 0.05, compared with the last breath; ^$^ *p *< 0.05, compared with the corresponding values in the pentobarbital groups.

There were considerable changes in breathing pattern during the period of profound hypothermia. In the pentobarbital group, the first breath on rewarming was significantly larger with a significantly longer *T*_I_ (Figures 5 and 6) than the last breath immediately before respiratory arrest (*p *< 0.05). On the other hand, there was no significant difference in the size of *V*_T_ between the first breath and the last breath in the sevoflurane‐continued group and the sevoflurane‐discontinued group (Figures 5 and 6).

It is also worthy to note that the values of *f*_R_ during the post‐RA period in the pentobarbital group were significantly greater than those in the sevoflurane‐continued group and the sevoflurane‐discontinued group, suggesting that the recovery of *f*_R_ immediately after respiratory arrest was much faster in pentobarbital groups than that in two other groups (Figure 6).

**FIGURE 7:**
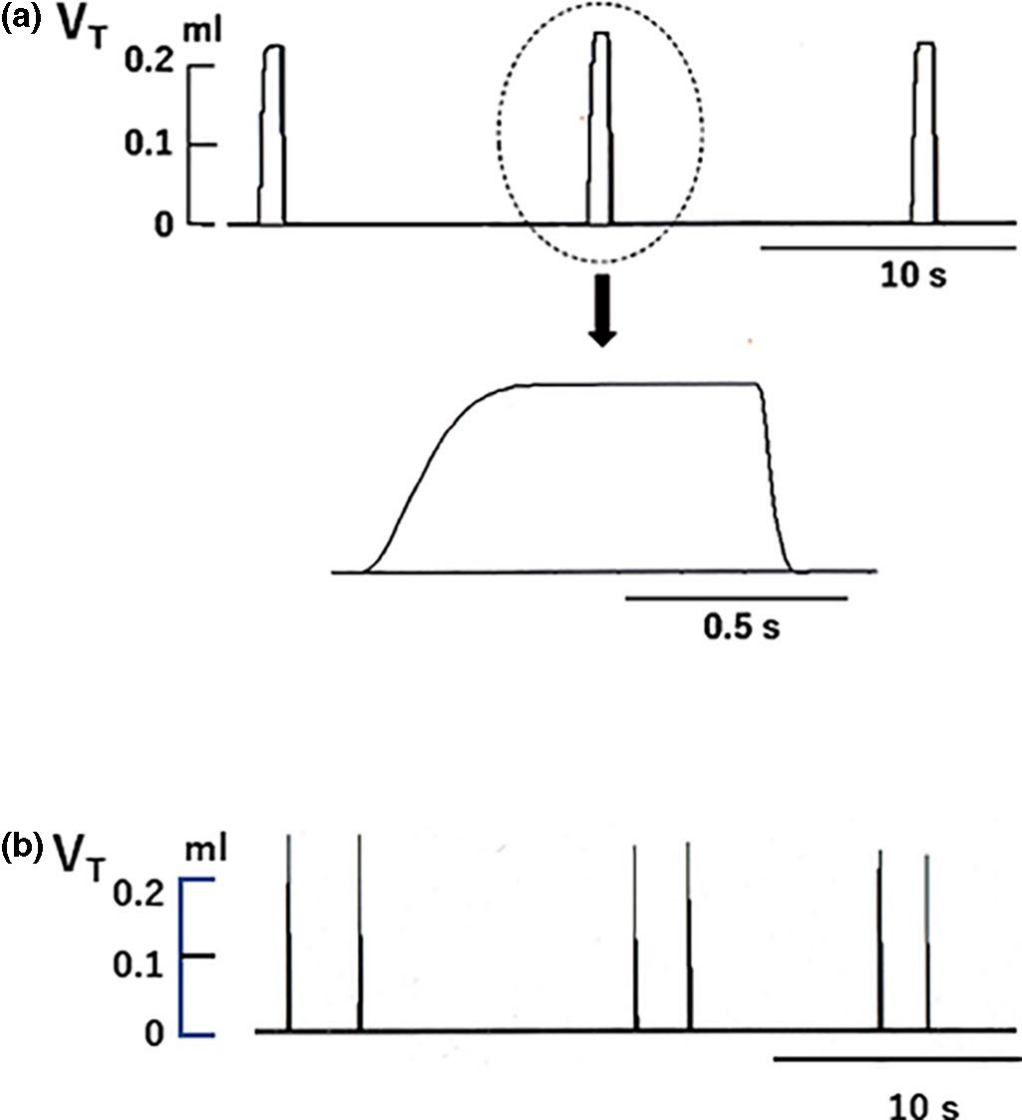
Apneustic breathing and episodic breathing observed in the sevoflurane‐discontinued group during profound hypothermia. (a) Apneustic breathing and (b) episodic breathing

In the sevoflurane‐discontinued group, a short period of apneusis and episodic breathing (Figure 7) were observed in two different animals immediately before respiratory arrest, whereas no such breathing patterns were observed in the other two groups.

During the post‐RA period the values of *V*_T_ were significantly greater than those of the last breath in both the sevoflurane‐discontinued group and the sevoflurane‐continued group, although the corresponding values of *V*_T_/*T*_I_ were significantly greater only in the sevoflurane‐discontinued group (Figure 6).

### 3.5 Survival rate and post‐experimental complications

All animals in all three groups recovered spontaneously from respiratory arrest. Thus, the survival rate was 100% (Table 1). After the experiment, one animal in the sevoflurane‐continued group showed swelling in the hind paw, which was probably due to slight burning during rewarming. This animal was immediately euthanized with a large dose of pentobarbital. In all other animals, no major complications were observed after the experiments and no animal showed any signs of respiratory distress or neural damage.

## 4 DISCUSSION

In this study, the effect on breathing during progressive hypothermia was studied in three different groups of mice. We found that in hypothermia, the body temperature at which respiratory arrest occurs is much lower in the non‐chemically anesthetized sevoflurane‐discontinued group than that in the anesthetized groups: the sevoflurane‐continued group and the pentobarbital group (Table 1). We also found that adult mice (C57BL6) have the ability to autoresuscitate from hypothermic respiratory arrest under the three different conditions, that is, under 1) pentobarbital anesthesia, 2) 1 MAC sevoflurane anesthesia, and 3) cold narcosis where the effects of sevoflurane anesthesia would have been eliminated. In addition, we found that there is a considerable difference in breathing patterns during progressive hypothermia between sevoflurane‐anesthetized mice and pentobarbital‐anesthetized mice. All of these findings are compatible with the notion that anesthesia and hypothermia exert synergistic effects on changes in breathing pattern and the occurrence of respiratory arrest.

### 4.1 Cold tolerance and autoresuscitation

In early studies (Adolph, 1948a, b; Adolph, 1951; Adolph & Goldstein, 1959), cold tolerance was defined as the cold temperature value that was lethal to a mammal. Once this critical temperature is reached, spontaneous breathing stops and is followed by cardiac arrest, and the animals ultimately die. In more recent studies, cold tolerance was defined as the body temperature at which respiratory arrest occurs and developmental aspects of cold tolerance were investigated in neonatal mammals (Corcoran et al., 2012; Tattersal & Milsom, 2003). The results of these studies show that the ability to autoresuscitate is lost in neonatal rats at postnatal days between 14 and 20. The results of our present study clearly showed that the use of anesthetic can influence the body temperature at which respiratory arrest occurs. If cold tolerance is defined as the temperature of respiratory arrest, in our study, it appears that anesthetized mice are less tolerant to cold temperature than unanesthetized mice. However, it is obvious that changes in temperature at which breathing stops are not linked to the ability to autoresuscitate since in all three groups of this study the rate of autoresuscitation is 100%, regardless of changes in cold tolerance. In this context, Corcoran et al. (2012) also noted that respiratory arrest need not lead to death and that the temperature at which breathing stops is not the same as the lethal temperature and does not necessarily reflect the full extent of cold tolerance of the animal.

### 4.2 Synergistic effects of hypothermia and anesthesia

Cooling without anesthesia is a common technique in hypothermic studies, but this method burdens the body with stressful loads. Thus, cooling without anesthesia might be a confounding factor that obscures the mechanisms of the effects of cooling on respiration. The present study used two different types of anesthetics, that is, pentobarbital and sevoflurane. Both anesthetics effectively eliminated cardiovascular, respiratory, and shivering responses, and resulted in smooth induction of hypothermia. Sevoflurane is particularly useful for providing the condition of cold narcosis since the sevoflurane can be easily removed once the body temperature is low enough (22ºC). Using a similar experimental method, Volkert and Musacchia (1976) have shown that light anesthesia with halothane not only reduces the biological insult during induction of hypothermia but also greatly enhances the survival of hamsters recovering from hypothermia.

In our study using anesthetized adult mice, we confirmed the previous observation seen in unanesthetized adult and juvenile rodents, that severe hypothermia causes a gradual decrease in respiratory activity and leads finally to respiratory arrest (Adolph & Goldstein, 1959; Corcoran et al., 2012). The high rate of survival (100%) after the respiratory arrest observed in all three groups in our study (Table 1) is compatible with the fact that the laboratory mice are daily torpor animals that behave like hibernating animals (Sunagawa & Takahashi, 2016; Swoap & Gutilla, 2009). Also, the observed high rate of survival may be related to the methods used in this study. First, using an endotracheal tube can provide the secure maintenance of an open airway and contribute to the prevention of hypoxemia during progressive hypothermia in spontaneously breathing animals. When the airway is patent, and the heart is beating, adequate oxygen uptake by the lungs may be possible even during respiratory arrest (Sullivan & Szewczak, 1998; Szewczak & Jackson, 1992). Second, anesthesia with pentobarbital or sevoflurane may exert multiple potential modes of neuroprotective actions, including reduced cerebral metabolic demand, antioxidant effects resulting in decreased cerebral edema, anticonvulsant effects, and preconditioning effects (Crane et al., 1978; Dzhala et al., 2008; Lin et al., 2014; Nilsson, 1971; Singh et al., 2004; Smith & Marque, 1976). Third, we performed our experiments under hyperoxia, which would minimize the occurrence of hypoxia during respiratory arrest and contribute to the retention of the intrinsic ability to autoresuscitate from hypothermia‐induced respiratory arrest.

### 4.3 Changes in breathing pattern

The considerable differences in breathing pattern between sevoflurane‐anesthetized mice and pentobarbital‐anesthetized mice could be attributed to different effects of these two different anesthetics on the central respiratory network including brainstem and supra‐pontine structures. It has been reported that neurons in the central respiratory network express GABA_A_ receptors (Brockhaus & Ballanyi, 1998; Shao & Feldman, 1997) and there is some evidence to show that respiratory depression by barbiturates is due to GABA_A_ receptor‐mediated inhibition, with the principal effects being an inhibition or slowing of central rhythm generation (Fregosi et al., 2004). Similarly, there is evidence to show that GABA_A_ receptors are the important functional sites of sevoflurane action (Stucke et al., 2005) in that GABA_A_ receptors in the pre‐Bőtzinger complex play a crucial role in sevoflurane‐induced respiratory depression (Kuribayashi et al., 2008). A combination of hypothermia and anesthetics, regardless of whether it is pentobarbital or sevoflurane, might similarly cause a change in breathing pattern characterized by a progressive decrease in *f*_R_ with little or no change in *V*_T_ during the early stage of cooling. Assuming that hypothermia‐induced respiratory depression may be a GABA‐mediated effect, the breathing pattern observed in pentobarbital‐anesthetized mice and sevoflurane‐anesthetized mice during this stage may be explained by the synergistic activation of GABA_A_ receptors although the effect of sevoflurane appeared to be much more than that of pentobarbital.

In our study, the values of *V*_I_ and *f*_R_ at 20˚C in the sevoflurane‐discontinued group were significantly higher than those in the sevoflurane‐continued group. These results are compatible with the idea that removal of sevoflurane causes attenuation of the synergic activation of GABA_A_ receptors due to both sevoflurane and hypothermia. However, the augmented breaths observed in sevoflurane‐anesthetized mice during the middle stage of cooling cannot be explicated by a simple activation of GABA_A_ receptors in the brainstem. The fact that in pentobarbital‐anesthetized mice there was a decrease in *V*_T_/*T*_I_ at 25ºC, whereas no such decrease in *V*_T_/*T*_I_ was observed in sevoflurane‐anesthetized mice. Figure 3 suggests that there are some mechanisms that counteract the suppressive effects of hypothermia on the central inspiratory drive in sevoflurane‐anesthetized mice. One possible explanation would be that sevoflurane can facilitate the activity of excitatory neurons within a spatially distributed brainstem network and thereby increase inspiratory drive. Indeed, Takita and Morimoto (2010) showed that in an in vitro newborn rat brainstem‐spinal cord preparation, sevoflurane has a hidden stimulant effect on the respiratory rhythm oscillators.

In this study, we observed that the first breath upon rewarming was larger than the last breath before respiratory arrest in pentobarbital‐anesthetized mice, whereas no such observation was made in sevoflurane‐anesthetized mice (Figures 5, 6). A similar observation in unanesthetized neonate rats has been reported by Tattersal and Milsom (2003). We also observed that the recovery of *f*_R_ from respiratory arrest was much faster in pentobarbital‐anesthetized mice than that in sevoflurane‐anesthetized mice (Figure 6). Although the mechanism that caused these differences in breathing pattern immediately after the resumption of respiration between pentobarbital anesthesia and sevoflurane anesthesia is unclear, it is possible that the synergistic effects of hypothermia and each anesthetic on the central rhythm‐generating network can be quite different.

The changes in the respiratory pattern during rewarming are nearly mirrored images of the changes observed during cooling in all three groups (Figure 3). Thus, although the data of the respiratory variables during rewarming showed much wide variability compared with the data during cooling, the effect of body temperature on the respiratory pattern during rewarming is essentially the same as the effect during cooling. This is compatible with the view that hypothermic respiratory arrest might be due to reversible failure in the neural network responsible for the generation and transmission of the respiratory drive (Mellen et al., 2002).

Regarding other types of abnormal breathing patterns during hypothermia, Bartlett (1955) showed that apneustic breathing frequently appears in cats and rabbits anesthetized lightly with pentobarbital during severe hypothermia. A similar apneustic breathing pattern has been observed in unanesthetized neonatal rats during severe hypothermia (Tattersal & Milsom, 2003). Contrary to the observation made in these studies, in our research, we never observed apneustic breathing during severe hypothermia in mice in the pentobarbital group nor in the sevoflurane‐continued group. However, apneustic breathing was occasionally observed in mice in the sevoflurane‐discontinued group during severe hypothermia (Figure 5). It is also worthy to note that the effect of pentobarbital on the breathing pattern during severe hypothermia might be influenced by the depth of pentobarbital anesthesia (Bartlett, 1955). In our study, a relatively large dose of pentobarbital (80 mg/kg, i.p.) was used and this could be a reason why we did not see the apneustic breathing during hypothermia in our pentobarbital‐anesthetized mice.

### 4.4 Methodological considerations and limitations of the study

Several problems related to data collection and data interpretation need to be discussed. First, we could not obtain sufficient data on cardiovascular responses to severe hypothermia except for the measurement of HR. It is worthy to note that the ECG amplitude was greatly reduced and mingled with background noise in several mice during severe hypothermia so that the occurrence of cardiac arrest could not be ruled out in these mice. However, all mice recovered completely from the severe hypothermia. Also, in this study we avoided performing any invasive procedures such as arterial blood pressure monitoring via catheterization and arterial blood gas sampling because invasive procedures might disrupt the effects of hypothermia and disturb a rapid, normal recovery from the hypothermic respiratory arrest. Tracheal intubation was the only invasive procedure used.

Second, in the sevoflurane‐discontinued group, although we assumed that at a body temperature of 22˚C or lower the condition of anesthesia was maintained by hypothermia alone which is designated as cold narcosis, we cannot eliminate the possibility that the residual effect of sevoflurane after discontinuation of sevoflurane might influence the temperature at which respiratory arrest occurred. However, we do believe that the concentration and effects of sevoflurane would have diminished quickly after discontinuation of its administration (Heavner, 2001). Also, it is possible that the concentration of sevoflurane might not have reached the 1 MAC level of anesthesia immediately after resumption of sevoflurane inhalation which might have influenced changes in the breathing pattern during rewarming.

Third, in the pentobarbital‐anesthetized group the animals were anesthetized with a relatively large initial dose of pentobarbital (80 mg/kg, i.p.) with no additional dose throughout the course of the experiment. It is quite possible that the level of anesthesia during the period of cooling might be different from that during the rewarming period. Thus, a simple comparison of the effects of different anesthetics at the same temperature during the rewarming period may not be entirely valid.

## 5 CONCLUSION

We showed that adult mice are capable of autoresuscitation from hypothermic respiratory arrest, regardless of the presence or type of anesthetic. We also showed that anesthesia increases the body temperature at which respiratory arrest occurs during progressive hypothermia. The type of anesthetic also affects the breathing pattern that occurs during progressive hypothermia leading to respiratory arrest.

## CONFLICT OF INTEREST

The authors declare no competing interest.

## AUTHOR CONTRIBUTIONS

T.N. conceived and designed the research. S.T., T.N., and H.J. performed the experiments. N.S, N.N‐T., and S.I. helped to formulate the study protocol. S.T., T.N., H.J., N.S., N. N‐T., and S.I. interpreted the results of experiments and approved the final version of the manuscript.
